# Relationship, Discourse and Construction: The Power Process and Environmental Impact of the Honghe Hani Rice Terraces as a World Heritage Site

**DOI:** 10.3390/ijerph192417100

**Published:** 2022-12-19

**Authors:** Honglian Hua, Yikun Wang, Zhiqiang Ding, Hua Liu, Shangyi Zhou, Yuli Liu

**Affiliations:** 1 Faculty of Geography, Yunnan Normal University, Kunming 650500, China; 2 School of Geographical Sciences, Fujian Normal University, Fuzhou 350007, China; 3 Faculty of Geography, Beijing Normal University, Beijing 100875, China; 4 Institute of Belt and Road, School of Business, Qingdao University, Qingdao 266061, China

**Keywords:** power, political ecology, environmental impact, World Heritage site, Honghe Hani Rice Terraces

## Abstract

The coexistence of conservation and degradation is a challenge for protected areas, and unequal political and social power is the mechanism underlying this conservation paradox. The World Heritage site of the Honghe Hani Rice Terraces (HHRT) has important natural and cultural value, but despite the enormous investment in protecting the site, the rice terraces continue to degrade, and much of the degradation has been unexpected. This study attempts to reveal the mechanism of these unintended protection outputs from the perspective of power relations. After reviewing the literature on the political ecology of protected areas, this study further considers the conceptual framework of power in view of the ambiguity of the concept and integrates the themes from research on protected areas into the power analysis framework of political ecology. Three aspects of the power process and environmental impact of heritage sites are analyzed: the actor network, conservation discourse and natural reconstruction. The results reveal that power among actors in the HHRT has changed over the course of continuous interaction, power has been produced and re-established in different relational networks, and the exercise of power has changed and reshaped the natural environment of the heritage site through a series of spatial planning decisions. Conservation discourse related to heritage is an important way for actors to establish and exercise power. However, due to spatial differences in the allocation of power, local development opportunities are unbalanced. In this unbalanced relationship, in order to maintain or strive for development opportunities and achieve economic development, residents of the HHRT have reshaped the natural environment by changing their farming methods and traditional planting methods, posing a potential threat to the sustainable development of the heritage site.

## 1. Introduction

The coexistence of conservation and degradation is a common contradiction in many conservation spaces. Van Schaik studied the protection of 201 national parks in 16 tropical countries and found that the development of nearly 70% of national parks deviates from the original protection goals, and the local human–environment interaction becomes increasingly tense during the process of protection [1]. Oldekop conducted an overall evaluation of the conservation effectiveness of 167 protected areas around the world based on the literature and found that most protected areas had unanticipated negative outcomes that affected their sustainable development [2]. Painter noted that previous explanations for the deterioration of the human–environment relationship in protected areas focused mainly on population growth, economic irrationality and backward management practices, which made it difficult to explain current problems in protected areas [3]. Paulson and Gezon pointed out that unequal political and social power is the underlying mechanism affecting ecological environmental changes [4]. Resource control is the purpose of the conservation policy of any protected area, and the conservation process is also a resource redistribution process. In this process, government protection policies often change the resource use rights of local communities, and local residents fail to benefit from resource protection, which eventually leads to unsupported protection actions or even the destruction of protected areas [5]. A heritage site is a complex space related to “development and environment, power and conflict” [6]. Therefore, research on ecological environmental changes in protected areas needs to focus on the dynamic changes and impacts of power networks [7].

Analyzing sources of power and their influence on ecological problems is the basic proposition of political ecology. Robbins argued that the power perspective in political ecology can effectively explain why many conservation projects that were initially considered effective turned out to be harmful or unhelpful or even failed [8]. He pointed out that current protection practices are usually implemented according to the “top-down” logic; in this process, local traditional and effective livelihoods and forms of social organization are disrupted by the official purpose of “protection”, which leads to many unintended consequences. Some studies have pointed out that there are three main reasons for these consequences. First, traditional resource management strategies are closely related to local institutional systems, and the imposition of protection policies often destroys those systems, leading to confusion about resource use and weakening people’s sense of responsibility for natural system management [9]. Second, conservation agencies undermine the integrity of traditional ecosystems by borrowing “scientific” concepts that result in a separation between humans and nature [8]. Third, the identification of protected space as a physical space with boundaries causes many problems at the level of ecological practice and social management. Botkin noted that the establishment of conservation space represents the imposition of political boundaries on ecological space, and conservation space boundaries will interfere with the integrity of ecological processes [10]. In addition, the macrolevel government management system is superimposed on the local social organization, and inconsistencies between the two will lead to injustice and conflict in the use and allocation of resources by various actors.

The Honghe Hani Rice Terraces (HHRT) are a World Cultural Landscape Heritage Site as well as a globally important agricultural cultural heritage pilot and national wetland park. In recent years, great efforts have been made to protect the HHRT, but many rice terraces in the core area continue to degrade [11,12]. Consistent with all conservation spaces, the HHRT World Heritage site was formed through the joint construction of many actors, whose different purposes, propositions and intentions are the underlying mechanisms that effect changes in the local human–environment relationship. As heritage sites enter the post-application era, the diversification of external actors makes the whole actor network more complex, increasing the uncertainty of the impact on the human–environment relationship. Therefore, to ensure the sustainable development of the HHRT, we must consider how external political and social powers reshape the local human–environment relationship and what the neglected consequences are.

Based on the above, this article focuses on the shaping of the power process and its impact on the ecological environment of the HHRT based on the theory of political ecology. For any conservation space, the power process is the result of the interaction of political, economic and cultural processes at different temporal and spatial scales. Therefore, the power relationship is a complex network, and the power process is elusive. To clarify the consideration of power in previous research on the political ecology of protected areas, we will review research on the political ecology of protected areas in the second part of this paper and summarize the “location” where power usually appears. In addition, as shown by the current diversified discussion of power in political ecology, the conceptualization of power has always been a focus of the field. In the third part of this study, the effective research conclusions on the current conceptualization of power will be presented to build the analysis framework of the HHRT. The fourth part shows in detail the power process of the HHRT and its environmental impact.

## 2. Review of the Literature on Political Ecology in Protected Areas

The deviation of protected areas from conservation goals has long been a challenge for conservation work. Previous studies have shown that “coercive” power in the process of conservation is a reason for poor protection. St. Martin’s study on reasons for the degradation of New England fishery reserves revealed that the logic of the “tragedy of the commons” cannot explain the degradation of fishing grounds and that incompatibility between the protection space forcibly demarcated by the government and people’s fishing habits and the living space of aquatic organisms is the problem [13]. Laney investigated the Baima Laha Nature Reserve in Madagascar and found that it had long been believed that forest degradation and declining biodiversity in the protected areas were caused by local people’s irrational slash-and-burn practices, and conservation organizations had not responded to local traditional environmental practices. The limitations of protection and the resulting conflicts with local people are reasons why it is difficult to implement protection [14]. Neumann concluded that protected space is basically a form of hegemonic regulation. For example, Arusha National Park in Tanzania was established by territorializing protected spaces with the eviction of local populations; and such power-based coercive management practices have been a major cause of land-use conflicts and the destruction of protected areas [5].

The abovementioned political ecology studies have pointed out the mandatory features of conservation and the ecological and environmental consequences, but this does not mean that political ecology is opposed to conservation. Political ecology hopes to determine the specific mechanism of environmental problems in these conservation spaces by analyzing failed conservation projects to negotiate the goals, aspirations and interests of different actors and realize the sustainable development of conservation space. From the perspective of the analytical logic of political ecology, the basic proposition is to oppose the dichotomy of natural processes and social processes. Therefore, the emphasis on the construction of nature by different actors in political and economic processes is the starting point of political ecology as a whole. The discussion of the power relationship behind the process is the focus of the research. Finally, the ecological environmental impact of the discourse, system and practice of the power subject is the foundation of the analysis and the starting point of a new approach. Following this basic logic, to convey power processes and environmental impacts in conservation, previous political ecology studies of protected areas have focused on three main aspects: the social construction of conservation space, the territorialization of conservation space and the protection/development discourse of power subjects.

### 2.1. The Social Construction of Conservation Space

The social construction of nature is based on the essential consensus that changes in environmental and ecological conditions are the result of social and political processes [15]. In many reserves, the social construction of nature is realized mainly through the discourse of scientific knowledge [16]. Scientific classifications such as “wilderness”, for example, reflect conservationists’ special vision of the environment and often refer to places without vegetation or a history of advanced agriculture. Sowerwine pointed out that in many countries in South and Southeast Asia, the concept of “wilderness” is used with similar social, political and ecological intentions: Land that has long been intensively grazed by local people, has lain fallow or has been used to collect firewood is often regarded as “wilderness” by the government. Economic and political power is applied to reclassify these lands as unproductive landscape types with the purpose of legitimizing resource possession and control [17]. Williams also showed that “wilderness” provides a rationale for the government to enter certain agricultural and pastoral areas and implement intensive grazing policies, and “wilderness” is spatially identified and protected with the purpose of building “fences” to carry out centralized animal husbandry production and remove obstacles to national economic growth. Such social construction based on scientific knowledge has a certain political mission [18]. Sowerwine further pointed out the ecological and environmental problems caused by the social construction of protected space in his research on forest protection in Vietnam [19]. His research showed that the entry of international conservation organizations and conservation capital has strengthened the legitimacy of government forestry reforms and forest conservation. However, in the process of forest classification and protection practices, the government’s “imagination” and division of forestland conflict with the habits of local residents. A large number of forest reserves designated by the government and conservation organizations are part of the land rotation of local residents, while the government forcibly requires afforestation. However, contrary to the official scientific classification of “wilderness”, these are not unproductive lands but rather places where local herbs are grown and biodiversity is abundant. The government’s classification thus “misreads” the local character, and the government promotes the planting of large numbers of exotic tree species, which ultimately destroys biodiversity in these “wastelands”. Moreover, since most local people depend on medicinal plants for their income, dwindling medicinal plant resources have led villagers to expand the cultivation and production of cassava elsewhere, exacerbating the destruction of forests.

### 2.2. The Territorialization of Conservation Space

The social construction of nature is performed by different actors. The power relationship between actors and the ecological problems that they generate are at the center of political ecology research [20]. “Power geometries” is the basic approach to analyzing the power relationship behind ecological issues [21]. Based on the dialectical and unified relationship between power and space, the power relationship is often presented through the process of territorialization, and the rationality of the designation of protective space boundaries is closely related to ecological and environmental issues. Sack believed that the demarcation of a specific spatial scope is a common means of implementing conservation strategies, that the territorialization of conservation space is based on power relations to demarcate the boundaries and implement the management of the space, and that this process is associated with specific intentions [22]. Peluso’s study in Kenya exemplified the essence of the territorialization of conservation space. She found that access to valuable resources and the commercial tourism development of rare resources are strategies for many African governments to increase their income [23]. When the government’s control of resources is disputed by local resource users, the government relies on its actual power to impose its will. With the rise of wildlife tourism, the government established national parks on the basis of hunting areas, demarcated the core area and buffer zone of a park, and established a large ranch to try to resettle local residents who moved out of the national park, resulting in severe restrictions on the livelihood of local residents. Harris and Hazen concluded that the delineation of many protective space boundaries is an attempt to include some people and exclude others. In the process of territorialization, the demarcation of protected space is affected by unequal power relations and differences in the ecological knowledge of different subjects; as a result, the determination of protection boundaries is often unreasonable [24,25]. The resistance of indigenous peoples in Indonesia to forest territorial space and the resistance of aborigines in eastern Niagara and southern Belize to forest protection space both prove the irrationality of territorialization [26,27]. This unreasonable territorialization process greatly challenges the sustainable use of natural resources [28]. Gillespie confirmed this view in his research on the Angkor Wat World Heritage Reserve [29]. His research showed that the World Heritage site involved multiple stakeholders, and the protection boundary of the heritage site was inconsistent with the local habitual agricultural production space, resulting in increased uncertainty of land use. Uncontrolled and varied agricultural production and tourism development facilities within the reserve resulted in the destruction of the landscape and environment of the heritage site.

### 2.3. Conservation Discourse

Power is connected to knowledge and discourse, and the production and operation of discourse are closely related to the purposes and intentions of the power subject [30]. For many conservation projects, it is difficult to achieve consistency in the ecological outcomes and social contributions produced by the protection discourses of power subjects at different scales and in different contexts. Therefore, it is necessary to examine the interpretation of the protection discourses produced by key actors and their impact on the ecological environment. Mirabzadeh-Ardakan investigated international wetland conservation and pointed out that the conservation discourse of the Convention on Wetlands is inconsistent at different scales [31]. On a global scale, the conservation discourse of the Ramsar Convention on Wetlands is “use wetlands wisely”. However, at the national level, the international conservation discourse is reflected only rhetorically and not substantively in the development discourse about the Alagol, Ulmagol, Ajigol (AUA) wetlands in Iran. The postrevolutionary Islamic government wants to improve its international image only by pandering to the international protection discourse rather than actually implementing protection projects. Controlling contested resources to achieve economic growth and rapid industrialization are the real goals. Therefore, the government considers the local people a potential threat to the alteration of the natural landscape of the wetlands. The livelihoods of local residents, equitable distribution of resources, and land-use conflicts are not taken seriously. On the grounds of protecting the AUA environment, the government has centralized control over land and local residents through forced settlement and land transfer policies and achieved economic growth through agricultural mechanization and industrialization. In the process, the pastures around the AUA wetlands have been diverted for dry crop cultivation, and agricultural irrigation cannot be practiced. Due to the lack of irrigation, local residents abandoned their customary land and water resource management models and sold the land for new land uses. The entry of new land users and the movement of local residents to obtain livelihoods elsewhere posed a potential threat of degradation of rangeland vegetation, landscapes and habitats.

## 3. Research Framework

Power is a broad and controversial concept that needs to be conceptualized to be analytically productive [4,32]. There are four conceptualizations of power in current political ecology research. The first is the “system” perspective of power, which draws on the neo-Marxist perspective in emphasizing that power is constrained and generated by the social structure established by history and mainly analyzes how power is exercised through economic domination and exploitation [33]. The second is the actor-oriented perspective of power. As power is not just a “resource” that is unequally distributed among humans but also a “capacity” to act or not act [32], actors are considered to exercise power through actions to achieve a specific intent. This perspective focuses on the process by which multiple actors exercise power and the outcomes of their negotiations. The third is the poststructuralist concept of power, which emphasizes the analysis of discourse and governance capacity [33]. In terms of discourse, it emphasizes how actors exercise power in a way that suits them by building a narrative of an issue, and in terms of governance, it emphasizes improving specific groups as “resources to be nurtured, harnessed, and optimized” [34]. The fourth is the constructive power perspective, emphasizing power as relational, situational, productive and contingent, providing space for interpretation of the ambiguous and contradictory outcomes caused by the exercise of power [35], and focusing on analyzing how new actors emerge from multiple and intersecting ways of exercising power in social networks [36]. Additionally, it considers how new actors shape the physical environment in the process of re-expressing, competing in and affirming power relations [37]. Among the four conceptualizations of power, the first is the understanding of power at the macrolevel and the other three are the understanding of power at the microlevel. These perspectives overlap, so power can be conceived of as a combination of these perspectives, although the weight of each type of power may vary depending on the actual situation.

Ahlborg and Nightingale pointed out that the concept of power is quite different in different fields [35]. For World Heritage sites, the overall goal is conservation, which requires the investment of funds, manpower and technology from government at all levels. However, in this process, the enormous investment is counterbalanced by the continuous degradation of heritage. How to reveal the mechanism of this unexpected protection output is the most difficult problem. The impact of external policies and the economic environment on the ecological and environmental problems of heritage sites is relatively direct and predictable. Therefore, performing an analysis from the “system” perspective of power is not the focus of our investigation. The process of nomination and management of World Heritage properties involves the joint participation of actors at different temporal and spatial scales. Mapping the exercise of power from the perspective of multiple actors can provide clues to various unexpected and unforeseen consequences beyond the exercise of power; therefore, an actor-oriented power perspective is central to the analysis of the political ecology of World Heritage sites. In addition, the exercise of actors’ power requires discourse resources so that actors can disseminate their views and actions on heritage protection in a way that is suitable for exercising power. Therefore, in this study, it is necessary to discuss the rights of actors with discourse power. The protection discourses of actors are analyzed in order to reveal the assumptions and claims in the discourse and the environmental processes that are influenced and dominated by them. Finally, to avoid the solidification of power-constituting subjects, which leaves no room for free agents, we also use the perspective of constitutive power to analyze the emergence of new subjects in accidental situations and how they reshape the natural material environment of the heritage site from the perspective of power relations.

**Figure 1:**
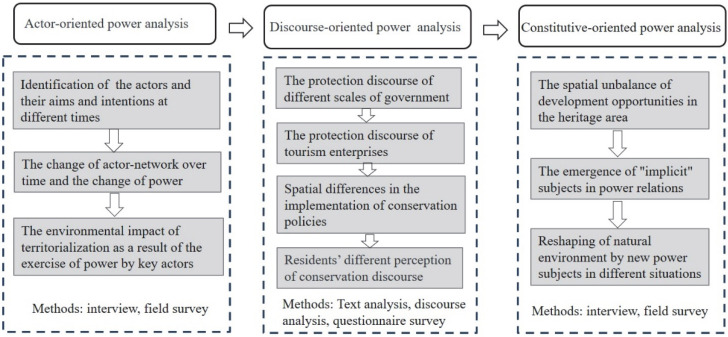
The research framework.

Based on the above discussion, we will present an inclusive research framework to analyze the power process and environmental impact of the HHRT. According to the current major trends in the conceptualization of power in political ecology, this paper analyzes the three main perspectives of actor orientation, discourse power and constitutive power while integrating the social construction of nature, territorialization process and development discourse issues from the literature of protected areas and aiming to conceptualize the issue of power at the HHRT (more details of the research framework are shown in Figure 1). First, we analyze the relevant actors and their target intentions since the HHRT was declared a World Heritage site to understand their basic actions in exercising power, and we analyze territorialization as the result of the exercise of power on the basis of previous research on protected areas. Second, we analyze the conservation discourses of key actors and emphasize discourse-based social construction in the literature on protected areas. The discourse analysis shows mainly how government agencies, enterprises and other actors exercise power and territorialization based on the discourse of heritage conservation management. Third, from the perspective of constituting power, we analyze imbalances in local society caused by key actors exercising power. In this process, local residents become new subjects in power relations. The analysis focuses on how local residents achieve balance in development by reshaping the local natural material environment in the new development context.

## 4. Power Process and Environmental Impact on the HHRT

**Figure 2:**
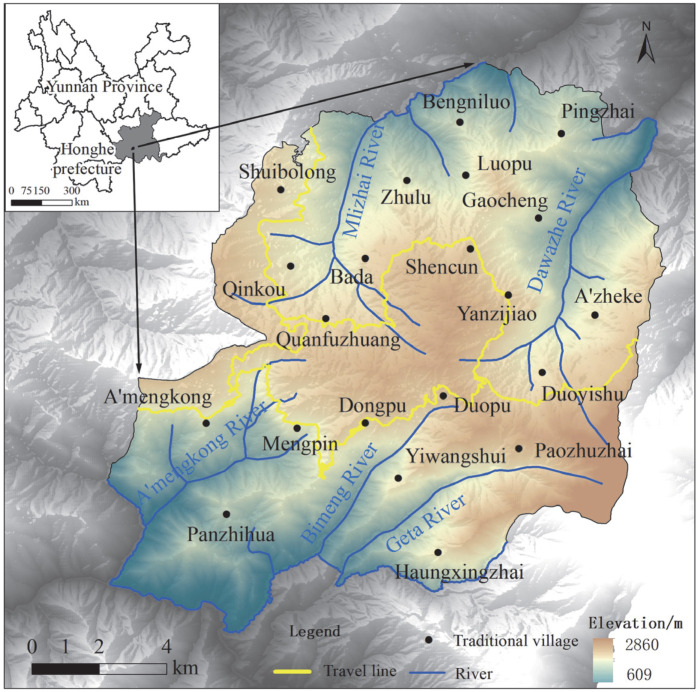
The location of the HHRT World Cultural Heritage site and the villages in the core area.

The HHRT World Heritage site is located in Yuanyang County, Honghe Prefecture, Yunnan Province, China, on the southern slope of the Ailao Mountains. In 2001, the process of applying for World Cultural Heritage status for the HHRT was officially launched, and local tourism activities developed rapidly because of extensive publicity. In 2008, with the support of tourism enterprises and the local government, a government-led, enterprise-participation, and market-operated tourism development model was implemented for the HHRT, and one tourist distribution center and two tourism service centers were built. In June 2013, the HHRT was officially listed as a World Cultural Heritage site, and the core area and buffer zone were designated. The core area contains three scenic spots: Bada, Laohuzui and Duoyishu. The whole heritage area contains 21 village committees and 85 natural villages; the geographical location of the HHRT World Cultural Heritage site and the village distribution in the core area are shown in Figure 2. These 85 natural villages are divided into three protection levels (8 villages under first-level protection, 51 villages under second-level protection and 26 villages under third-level protection) to maintain the intrinsic sustainable power of heritage. Since the area became a heritage site, the overall transportation system has been greatly improved, tourism development has become more rapid, and the demand for the development and utilization of the heritage site has reached an unprecedented level. At the same time, the degradation of the HHRT, mainly in areas with intensive tourism development activities, has been very significant, and the rice terraces show a decreasing trend. Unexpectedly, areas with low levels of tourism development are experiencing the degradation of rice terraces to dry land for growing cash crops. To understand the mechanism behind this process, from 2013 to 2020, we conducted research and surveys on heritage sites, involving actors such as the Hani elites, government agencies at all levels, expert groups, tourism companies and local residents. Materials research involved the interpretation of heritage declaration texts and heritage management regulations at different scales. Field research was conducted on many occasions, and 6 experts were interviewed 15 times in total. Three estate administrators were interviewed 5 times each; 2 managers of tourism enterprises were interviewed 6 times each; and more than 120 local residents, covering 10 village committees, were interviewed 17 times each.

### 4.1. Power in Actor Networks

#### 4.1.1. Actor-Network Construction and Power in the Heritage Declaration Period

From 2001, when the HHRT was officially declared a World Cultural Heritage site, to June 2013, when the HHRT officially became a World Cultural Heritage site, the human actors involved in the construction of the world heritage of the HHRT included the World Heritage Committee, Hani elites, various government agencies, expert groups, tourism companies, tourists and local residents. Nonhuman actors included the unique natural landscape, natural conditions, natural resources, and cultural resources. In this process, two well-educated and socially influential Hani elites were key actors. They believed that if the HHRT were declared a heritage site, it would fall under the international protection category in the development strategy of the country, and they could eliminate the pattern of remoteness and backwardness, obtain national and international development opportunities and enhance national self-confidence.

The Hani elites tried to express “Honghe Hani” as a harmonious multiethnic whole, and their efforts received a positive response and support from the government. The main purpose was to meet the UNESCO World Heritage standards and successfully add the HHRT to the World Heritage list. Because the Philippines had one rice terrace listed as a World Heritage site before the HHRT, it was difficult to declare the same type of heritage site. Considering that the rice terraces in the Philippines were controversial because they did not take into account ethnic groups other than the Ifugao, the Hani elite, based on the concept of unity, advocated applying for World Heritage status under the name “Honghe Hani Rice Terraces”, referring to rice terraces reclaimed and cultivated by various ethnic groups in Honghe Prefecture, represented by the Hani people. The related water sources, forests, irrigation systems and ethnic villages were treated as cultural landscape areas. The name implies not only harmony among the Hani but also solidarity with other peoples across regional borders. The government was so pleased with this suggestion that the Hani elites gained its support for the project.

Under the propaganda and promotion of the Hani elites, the local government, tourism enterprises, experts and scholars, natural landscapes and local residents actively entered the network of actors and formed a common interest. Local governments can use the World Heritage designation to develop tourism and to promote local economic development. Tourism enterprises can attract more tourists and earn more economic income through the attractiveness of the World Heritage symbolic capital. Local residents can gain more employment and economic opportunities in a heritage site. After the HHRT landscape became a heritage site, its value was more widely understood and accepted. After a 13-year process of application for the World Heritage site designation, based on the World Heritage Committee’s evaluation criteria, a network of actors was established, led by the Hani elites, and the HHRT was transformed from an obscure and remote part of the countryside to a world-famous World Heritage site.

#### 4.1.2. Power in the Fission of the Actor-Network after Successful Heritage Declaration

After the heritage application succeeded, the multiple goals of various actors were clearly differentiated. Coupled with the World Heritage Committee’s romanticized concept of local ecological protection, this made it difficult to coordinate the World Heritage conservation goals and the intention of local economic development, which became the most serious problem in the whole network of actors. To cater to the evaluation system of the World Heritage Committee during the application stage, the Hani elites, government at all levels and expert groups positioned the Hani people as the original ecological people. In the process, the need to “pander” to global protectionist expectations made it necessary for these actors to adapt to the standards and discourses of the protection of World Heritage, especially rigid adherence to the principles of authenticity and integrity, which limits possibilities for economic development. The underlying intention of the local government in applying for the heritage designation was to obtain opportunities for local economic development from the World Heritage site, but the original ecological positioning suppressed opportunities to use the space for economic activities. To balance the conservation orientation with the urgent needs of local economic development, ecotourism became the first choice for local economic development. Since 2000, two traditional villages in the heritage site have become folk tourism villages, but ecotourism in these two villages cannot drive local economic development in the overall HHRT. Later, the government of Honghe Prefecture brought in the Yunnan Expo Group to improve infrastructure and develop tourism operations. Ticket collection was the main source of income. However, since tourism enterprises did not develop a consistent plan that involved local residents in terms of employment and dividends, the introduction of tourism enterprises failed to rapidly promote local economic development. At the same time, due to the backward tourism infrastructure and the low quality of tourism services such as interpretation and tour guides, the overall satisfaction of tourists was not high, and the value of the heritage site was not widely recognized and publicized. Therefore, the government’s primary goal of promoting local economic growth was inconsistent with the advocacy of the Hani elites to promote a spirit of nationalism and enhance national cultural confidence, and the Hani elites were eventually excluded from the network of actors.

#### 4.1.3. Power of Actor-Network Reorganization in the Post-heritage Era

After the success of the heritage declaration, tourism enterprises and real estate developers became key actors in the actor network, and the positions of expert groups, local residents, and terraced landscapes in the action network were constantly marginalized. When the HHRT became a World Heritage site, its protection and development were legally guaranteed. The local government, as the direct manager of the heritage site, obtained the legitimacy to protect and develop the heritage site, and the Honghe Prefecture World Heritage Administration and Yuanyang County Rice Terraces Administration became the main departments with responsibility for maintaining and managing the terraces landscape on behalf of the government. At the same time, to promote the local economy with the help of heritage, more tourism enterprises and real estate developers were recruited, and with the support of the local government, they gained the power of economic development and became key actors in the new actor-network. These actors also recruited nonhuman actors, such as local residents’ land, water sources, and cultural landscapes, into the actor network. Most local residents only passively provided land, water sources and terraced landscapes in this process. Although some residents have benefited from heritage tourism by opening family hotels, others have no way to substantially participate and have gradually been marginalized. In addition, the expert group completed the preparation of the text during the declaration period but then gradually withdrew from the actor network after the declaration succeeded.

**Figure 3:**
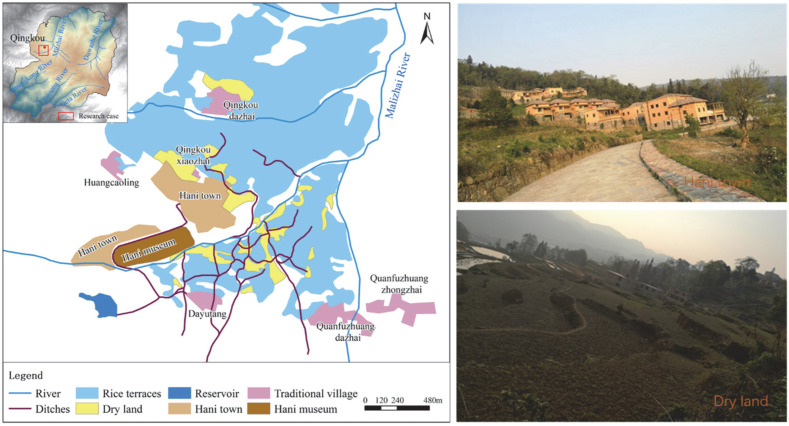
The locations of Hani Town, Hani Museum and the dry land.

With the establishment of the power status of the government and developers, a series of local shaping efforts gradually unfolded. In 2013, the government, promoting the country’s “Border Opening and Pilot Project” and “Beautiful Homeland Project”, introduced the Honghe Prefecture Tourism Investment Company with funding of RMB 220 million, and the Honghe Construction Group built “Hani Town” in the heritage site. The construction of “Hani Town” started on October 16, 2013, and it was completed and started operation in October 2014. It was one of the state-level “Beautiful Homeland” demonstration villages in Honghe Prefecture in 2013 and was selected for the second group of “Villages with Chinese Ethnic Characteristics”. In 2016, the Yuanyang County Bureau of Culture, Sports, Radio and Television commissioned Yunnan Architectural Engineering Design Institute (Group) Co., Ltd. to start the construction of the “Honghe Hani Terraced Fields World Cultural Heritage Management Exhibition Centre (Hani Museum)”. Hani Town and Hani Museum are located near a famous scenic spot in the core area of the heritage site that has an excellent visual effect. They cover an area of 100 ha., and the land used came from woodlands and tea gardens. A total of 221 buildings were built and sold mainly for tourism uses. The constructed spaces of Hani Town and Hani Museum are located on top of the three first-class protected villages in the heritage site. Historically, the water sources of these three villages have come from the same forest, and they have jointly negotiated and allocated water resources and cooperated in rice production. Hani Town draws water directly from the common water source of the three villages, which has caused the terraces around the three villages to gradually dry out due to a lack of water over the past five years. Coupled with the damage caused to some ditches and terraces by the construction of Hani Town, approximately 4. 7 ha. of surrounding terraced fields have been turned into dry land. The location of Hani Town and the dry land are shown in Figure 3.

**Figure 4:**
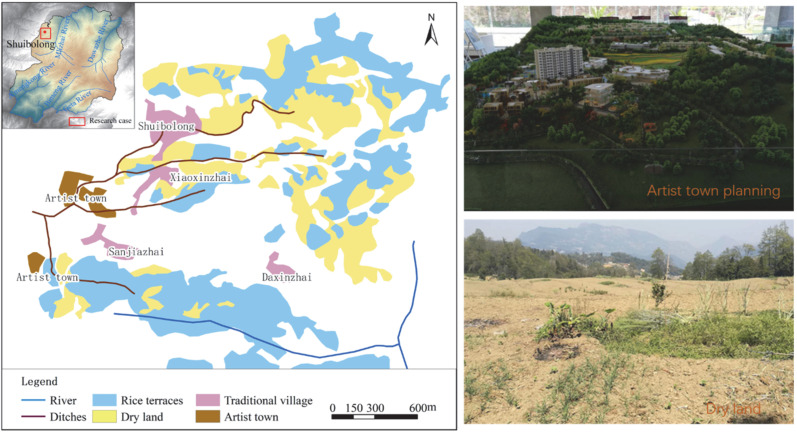
The locations of Artist town and the dry land.

To promote the World Heritage site and improve the tourism service level, as well as to enable more tourists to obtain an in-depth understanding of the HHRT and promote local development, in 2014, the Honghe Prefecture government arranged for Kunming Urban Construction Co., Ltd. and Yuanyang Land Investment Company to develop the “Artist Village” real estate project. With the influx of tourists into the heritage area, the numerous hotel business owners operating tours have had a negative impact on the rice terraces. The Artist Village is close to the entrance of the scenic spot and covers an area of 12.8 ha. The land used came from the former site of the Yuanyang County Canning Factory. The water used by the Yuanyang Canned Food Factory came from the important water source of Shuibulong Village. In 1985, the cannery signed a water transfer agreement with Shuibulong Village. The two parties reached an agreement to “transfer the water source to the cannery for use with compensation” and clearly stipulated that “after the water source is transferred to the canning factory, the tail water of the canning factory still flows back from the original channel and is used by the villagers, and no one shall interfere.” A total of 42 households were affected by this water transfer, involving a rice terrace area of 4.3 ha. In 1996, Yuanyang Canned Food Factory went bankrupt, and the original factory stopped production. In 2014, Landmark Investment Company developed the Artist Village on the former site of the factory. For its construction and operation, the Artist Village continues to use water sources from nearby villages—Sanjiazhai, Xiaoxinzhai, Shuibulong and Daxinzhai. The location of the Artist Village at the site of the original Yuanyang County Cannery, upstream from the four villages, has long cut off the water supply to the Changtian Ditch. Therefore, the terraces in the four villages are drying out to varying degrees. By 2020, a total of 15.2 ha of rice terraces in the four villages had become dry land. The locations of the Artist Town and the dry land are shown in Figure 4.

### 4.2. Protection Discourse and Local Interpretation

#### 4.2.1. Protection Discourse of Key Actors

“Standardized management” and “sustainable use” of heritage are important discourses for the conservation and management of heritage by the World Heritage Committee. Regarding the standard management of heritage sites, the World Heritage Committee clearly stipulates that a heritage site must have effective boundaries, there should be appropriate management planning and protection measures, and the conservation status of the heritage site needs to be assessed regularly. The sustainable use of the ecology and culture of the heritage site is clearly proposed to improve the quality of life of the community and to promote and encourage the active participation of the community and all stakeholders.

“Protection first, unified planning, scientific management and rational utilization” are the basic words used by national and local governments to implement the World Heritage Convention. Around this discourse, the local government issued a series of management regulations to echo the “management norms” of the World Heritage Committee. Among them, the Protection and Management Plan of the HHRT clearly defines the boundary between the core area and buffer zone of the heritage site and presents clear plans for land utilization, tourism development and environmental remediation within the heritage space. The Measures for the Protection and Management of the HHRT of Honghe Prefecture and Regulations on the Protection and Management of the HHRT of Yunnan Province define the specific management system and protection measures. In addition, based on the World Heritage Committee’s “sustainable utilization” discourse, the local government believes that heritage protection and tourism development can promote each other, and ecotourism is the main type of sustainable utilization. In addition, the Development Plan of HHRT Ecological Tourism and the Special Plan of Interpretation and Display System of HHRT have been formulated particularly for heritage tourism. In September 2014, to further improve the setting of protection and management institutions and increase investment in the protection of the HHRT, the government issued the Decision on Strengthening the Protection and Management of the HHRT, a World Heritage site, and set up a management committee in Yuanyang. Since 2014, the state government has allocated RMB 10 million every year as special funds for the HHRT.

“Integration, integrated development, building the community into a first-class scenic spot, and building the scenic spot into a harmonious community” is the ideal discourse for tourism enterprises trying to sustain heritage protection when carrying out heritage tourism based on local ecology and culture. Tourism enterprises believe that the HHRT is both a community and a scenic spot, and the two are inseparable. The development goal of tourism enterprises is to integrate the development of communities and scenic spots, enterprises and residents as well as visitors. By establishing an “innovative community-based scenic spot management model”, enterprises, as the development subject, guide and help villages to participate in protection and development, thus benefiting villagers. In the construction of scenic spots, tourism enterprises mainly strengthen the construction of tourism infrastructure in scenic spots. In terms of community support, according to the agreement, tourism enterprises pay 10% of ticket revenues to the six villagers in the core area of the terraces as well as sanitation and cleaning fees of RMB 2000 each. In each scenic spot, farmers who have acquired land will be assigned a business site of 0.5 sq. m. per household, which will be under the unified and independent management of the village.

#### 4.2.2. Local Residents’ Understanding of the Protection Discourse

Local residents’ understanding of the World Heritage protection discourse is obtained mainly from various measures of the local government and interaction with tourism enterprises. Local residents themselves do not have a clear concept of World Heritage. Their understanding of heritage sites is based mainly on the demarcation of heritage protection space and the implementation of relevant protection and management policies as well as their perceptions after the entry of tourism companies and tourists. In this process, the opportunities and changes experienced by local residents differ, especially given the division of the spatial boundary of the heritage site and the developed area. Additionally, the understanding and perception of local residents of the discourse and practice of heritage protection and development are spatially different. To more accurately reflect local residents’ perception of the government’s and enterprises’ discourse on heritage protection, this part of the data and information was obtained mainly through questionnaires and interviews. Representatives of villages affected to different degrees by government policies and enterprise tourism development were selected for analysis. The five villages were Qingkou, Dashang, Quanfuzhuang, Gaocheng and BaDa. Among them, Qingkou and Dayutang villages are located on the tourism line and listed as ecotourism folk villages by the local government; thus, they benefit the most from tourism in the heritage area. Quanfuzhuang and Bada villages are also on the tourism loop; However, they have no tourism development projects, representing villages with good transportation conditions and potential development opportunities in the heritage area. Gaocheng village is far from the tourism loop, traffic is blocked, and almost no tourists visit there, so it has almost no tourism income. A total of 120 people were surveyed in 5 villages; 18 in Qingkou, 16 in Yudang, 44 in Quanfuzhuang, 17 in Gaocheng and 25 in BaDa. The specific contents of the questionaire are shown in Table 1; It contained 9 questions scored on a five-point Likert scale. “Strongly agree, agree, generally agree, disagree and completely disagree” corresponded to 5 points, 4 points, 3 points, 2 points and 1 point, respectively. The score of each item is the average score of all the respondents. The scores of each village are shown in Table 1.

In the first category are Qingkou and Dayutang, which are important tourist villages that have been greatly affected by tourism development. These two natural villages are the most profitable in the heritage area. Regarding the government’s protection and management of rice terraces, villages, ditches, forests and traditional culture, the villagers in Dayutang are basically positive and satisfied, while those in Qingkou are basically satisfied with the government’s protection of terraced fields and with the renovation of the village but find the protection of ditches, forests and traditional culture basically unsatisfactory. However, residents of both villages agree that after the HHRT became a heritage site, their economic income and job opportunities improved. They believe that their contributions to heritage protection and their due compensation (income) are worthwhile. They are satisfied with the management of the integrated development of community and scenic spots by tourism enterprises, and the people of Qingkou are especially satisfied with this integrated development mode.

The villages in the second category, Quanfuzhuang and Bada, are located on the tourist line and are known for their scenic spots. Quanfuzhuang is located on the side of the road, and tourism enterprises have a viewpoint at the edge of the village, which makes it easy for tourists to enter the village to take advantage of a small number of catering and accommodation services. There are also viewing points in Bada, but the village is not as near the tourist route as Quanfuzhuang, and it is difficult for tourists to enter it. Residents of Quanfuzhuang and Bada feel that the government still attaches great importance to the management and protection of rice terraces, ditches and forests, but they think that government protection of traditional customs is insufficient. Regarding the government’s reconstruction of villages, since Quanfuzhuang was one of the first to be included in the traditional village protection and development plan, the villagers were quite satisfied with the government’s protection and reconstruction. However, the residents of Bada, because it was not included in the initial protection plan, are not satisfied. In contrast to Qingkou and Dayutang, they were not among the folk villages built by tourism companies. They feel that after the HHRT became a heritage site, their income and job opportunities did not change, and they are not satisfied with the promotion of local economic development by the government. There has been no equal return for efforts to protect terraced fields, and they were basically dissatisfied with the integrated development of communities and scenic spots promoted by the tourism enterprises.

Gaocheng is the third type of village; it is blocked by traffic, has no tourist facilities and is difficult for tourists to enter. Heritage protection measures have basically not affected the village, and almost no tourists visit it. The residents of Gaocheng believe that the government pays no attention to their rice terrace fields or to the protection of water ditches and forests, and they are extremely dissatisfied with the maintenance and renovation of their village and the protection of traditional culture. They believe that the heritage designation has not brought them more job opportunities and benefits, and their economic income has basically not improved. They express the view that they did not receive an equivalent return for their efforts to protect terraces, and they are extremely dissatisfied with the development mode of tourism enterprises.

**Figure 5:**
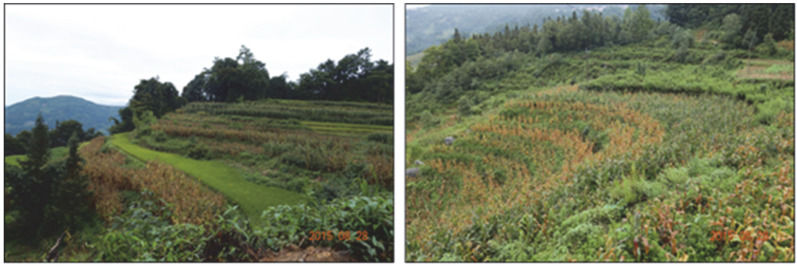
The rice terraces shift to land for growing corn in Gaocheng village.

The director of the Gaocheng Village Committee said, “The Rice Terraces Administration is an empty shell. It has never looked into whether we have irrigation water for farming. They have never visited our village for a day. Although we cannot develop tourism to make money, can they help us repair the ditches? This is not very demanding; even if you don’t come down yourself, you can give us some cement to repair the ditches by ourselves; we will be very happy. They’re all about the rice terraces nearby the travel line; they’re all about the rice terraces they can see. We’re down there, and tourists can’t see us, so they don’t care about us. Some fields are dry, and only when they are completely dry will the Rice Terraces Administration take care of us. At present, we can only grow some corn, and we can’t make much money. Now I hope the government arranges for agricultural talent to come to us to see what we can grow and improve our economy. In the past, the government encouraged us to plant red rice (a local variety), but there is no guarantee, and there is no purchase. The production of red rice is low. If the government does not come to collect it, the farmers will not even have enough food, and the risk is too great”. The rice terraces are shifting to land used to grow corn, as shown in Figure 5.

### 4.3. Nature Reconstruction by Local Residents

The spatial differences between the government’s protection and management measures and the tourism enterprises’ protection and development practices have led to spatial differences in local residents’ perceptions of the government’s and tourism enterprises’ protection and management attitudes, which further affects local residents’ different attitudes towards, and practices related to heritage. As mentioned above, local residents’ perceptions and understanding of World Heritage sites have been established largely through the implementation of relevant measures by the government and tourism enterprises. Such differences in perception have led local residents to adopt different ways of living, which will have a random impact on the heritage site.

For villages that are heavily affected by tourism development, such as Qingkou and Dayutang, the World Heritage designation is considered to be a shortcut for their economic development and employment, and attracting tourists has become their motivation for action. However, the residents of a poor community located in a rural area in the mountains have little capacity to act and must adapt their traditional habits to accommodate these powerful outsiders. Before the Expo Company took over the management of Qingkou and Dayutang folk villages, the villages were managed by the Yuanyang County Tourism Management Committee. Because 30% of the ticket revenue belonged to the villagers, they were highly motivated to participate. To attract more tourists year-round, Qingkou folk village changed its original farming system. In the HHRT area, the seasonal rotation of the terraces plays a very important role in maintaining their physical structure and sustainable use. The water in the terraces needs to be drained before seedlings can be transplanted and during harvesting and land preparation; otherwise, they need to be soaked. However, to ensure that the rice terraces are underwater year-round to attract tourists and photography enthusiasts, in Qingkou Village, local residents changed the seasonal rotation of the terraced fields to submerged farming year-round to provide a spectacular terraced field view that will attract tourists at all times of the year. The perennial immersion farming method requires more water sources for irrigation, so more water enters the rice terrace fields with a good view, while the farmers have difficulty maintaining irrigation for terraces outside the line of sight. These changes in farming patterns and the potential threats to local sustainable development have not been thoroughly considered by the local population.

Migu, a traditional village leader from Qingkou village, said, “As long as the government compensates us for the damage caused by the perennial soaking, it is worth it to soak the terraces all year round. If that happens, we can be sure that farmers will be more proactive in planting trees to ensure that there is enough water to fill their terraces, which is good for the environment”.

Villages near the tourism line, such as Quanfuzhuang and Bada, have begun to feel the effects of the government’s protection measures in maintaining terraced fields. Although tourism is not currently as profitable for them as it is for Qingkou, they recognize the importance of terraced fields for their future development, so they have adopted a wait-and-see attitude and will decide how to cultivate their terraced fields by watching the development trend. They will do their best to maintain terrace production.

**Figure 6:**
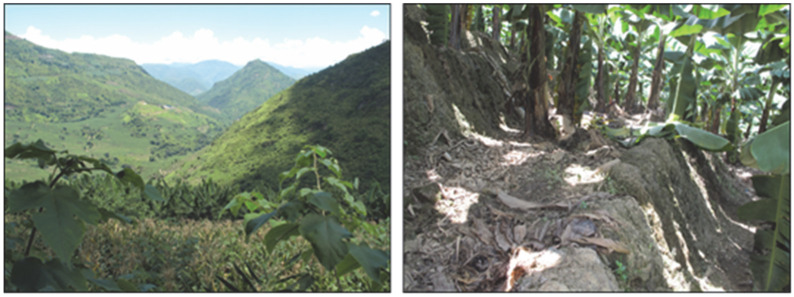
The shift from rice terraces to land for growing bananas in Gaocheng village.

It is difficult to balance the mentality of villages such as Gaocheng, which are inconvenient, closed, and have no tourism projects, with that of other villages that benefit. They have become more radical in their efforts to obtain development. They believe that the government cares only about villages that are located on the edge of the tourism loop and does not care about villages that are invisible to tourists. To gain economic income, they can only cut down and sell the fir forests that were planted on the barren hills during afforestation. In addition, due to the good economic benefits of fir trees, many dry terraced fields have also been planted with fir trees, and some villagers have cut down large areas of the artificial forest to plant citrus. Some downstream villages, due to good natural heat conditions, have leased a large number of terraced fields to contractors who grow bananas. Due to the considerable rent, they have given up rice cultivation. Interviews with local residents revealed their attitudes towards the government protection discourse and their desire for economic development:

“Planting bananas is very cost-effective. The owner who rents rice terrace fields gives us cereal, and they use the rice terrace fields to grow bananas. We only need to help them repair the ditch once a year. In addition, we help them manage bananas; RMB 7 per banana tree is the annual management fee. Our farm has been contracted for more than 4 years, and bananas can be bought for 5–6 years once planted. In these few years, we don’t have to worry about life”. The shift from rice terraces to land for growing bananas is shown in Figure 6.

## 5. Discussion

Applying the four conceptual directions of power from the current political ecology literature to research on the HHRT World Heritage site is fruitful because it not only integrates the themes of concern in previous research on protected areas but also contributes to exploring the ambiguous outputs caused by actors’ power relations. These unexpected ambiguous outputs are the real issues that affect the sustainable development of heritage sites. It should be noted that in the special context of China, among the four conceptualizations of power, the analytical path of systemic power should be used with caution. Especially in the protected area studies mentioned above, a large body of literature describes the oppositional relationship between the government and farmers in postcolonial settings, but the domination/resistance pattern does not fully correspond to the power relationship between today’s actors [38]. Especially in China, considering the issue of eco-environmental protection and the associated power relations in a framework of state/peasant confrontation misses the crux of the problem. People in China generally believe that the land belongs to the state, and there is an expectation of returns to the state for use of the land. Under such a concept, farmers’ views of collective land are not actually exclusive, as they believe the state has use rights [39]. Therefore, the analysis of power relations should be based more on how the state, capital and peasants negotiate than on following the opposition and resistance trend of Western studies.

The three power perspectives of actor, discourse power and construction effectively reveal the process and reasons for the emergence of the current protection paradox of the HHRT World Heritage site. However, it should be noted that current research on protected areas overemphasizes the use of power and the acquisition and control of resources while paying insufficient attention to ecological environmental changes. This should be avoided for developing countries that urgently need to address real-world environmental problems. As early as 1999, Vayda and Walker pointed out that the influence of politics on ecological environmental change is undoubtedly very important, but many studies have focused more on the power process of resource acquisition than on ecological environmental change [40,41]. Turner pointed out that the integration of a wide range of political, economic and ecological processes at the local scale is the most unique aspect of political ecology when applied to human-environment relations [42]. This analysis will lead to the erosion of political ecological theory, which will lose its methodological advantage in studying ecological processes based on specific local geographic and historical contexts. From the perspective of the development process of political ecology, it is an inclusive concept, and Western researchers have paid much attention to social and political aspects. However, for developing countries with many environmental problems, the focus of research should be biased towards the biophysical processes and environmental feedback.

## 6. Conclusions

The analysis of the actions of actors at different spatiotemporal scales revealed that the HHRT World Heritage site has always been a space in which many actors interact with each other, and their different aims, opinions and intentions, as well as changes in network relationships, are the dynamic mechanisms that shape the landscape of the heritage site. In the process of applying for World Heritage status, the World Heritage Committee, Hani elites, government at all levels, expert groups, tourism enterprises, tourists, local residents and local landscapes constituted a network of actors. However, the greatest contradiction in this network was that it was difficult to coordinate the protection goal of World Heritage designation with local governments’ intentions to develop the economy. When the HHRT officially became a World Heritage site, the government’s right to protect, develop and manage the rice terraces was legalized. As a result, the urgent economic development needs of the government diverged from the goals of the Hani elites representing the national cultural renaissance, and the Hani elites were excluded from the network. At the same time, conscripted by the government, real estate developers and tourism enterprises become the key actors in the actor network after the heritage designation was successful. Expert groups, local residents and the terraced landscapes were increasingly marginalized in the action network. A series of heritage development projects resulted in the occupation of rice terraces, inadequate water supplies and the destruction of rice terraces. Throughout the process, power has been embodied in what Gaventa described as follows: “Power can be understood as a network of social boundaries delineating possible fields of action”, and “power relations shape the boundaries of participatory spaces” [43]. After the successful designation of the heritage site, the social network space created by government at all levels from the state to local government and tourism enterprises as power subjects became a “closed space” [43], and local residents basically had no channels to participate in it. In this closed space, power is manifested in two forms: visible power and hidden power. As visible power, a series of planning and development measures carried out by the government and enterprises through territorialization has led to the occupation and grabbing of land and water sources, resulting in the transformation of rice terraces to dry land owing to a lack of water sources for irrigation. This process is observable, and its impact is intuitive and relatively easy to solve. Although its consequences are reflected indirectly, it is a potential threat to the sustainable development of the heritage site.

Government at all levels and enterprises, through the enactment of relevant protection laws and regulations, create the discourse of protection as an expression of their hidden power, which is an important way for key actors to further establish their power. In the process of power allocation, spatial differences are created, which lead to the imbalance of development opportunities within the heritage site. The spatial imbalance in the exercise of power exacerbates the social imbalance. Local residents are directly related to the unbalanced development opportunities in heritage areas. To maintain or strive for development opportunities and obtain economic development, local residents try to break through the existing restrictions of power relations in a self-interested way, and their subjectivity is gradually generated in these multiple and overlapping power exercises. This creates a series of “created spaces” in the form of “implicit power” and shapes the natural material environment with hidden practices. For example, by changing the traditional farming system, farmers soak the rice terraces with water for years to attract tourists, which is not conducive to the sustainable development of the terraces. Villages on the tourism ring line, although tourism has not yet produced significant profits, believe there are still opportunities, so they continue to cultivate rice terraces and adopt a wait-and-see attitude. Villages with blocked traffic and no tourism projects take a more radical approach to obtain development. They cut down forests to obtain income by obtaining wood, and they plant fruit trees and other cash crops in terraced fields on the grounds that there is no water for irrigation. These practices have become their way of breaking through the existing “closed space” and trying to establish their own subjectivity in power relations.

Based on the above three perspectives, actor orientation, discourse orientation and constitutive orientation, we can clearly analyze the power process of the HHHRT, and identify the nature, performance and influence of power at different stages. This analytical framework is applicable to the specific context of China. It avoids the control—resistance binary opposition commonly used in the study of power, especially the constitutive-oriented perspective, and highlights power relations as both repressive and productive. Productive thought helps us to draw closer to the dynamic nature of power relations without immobilizing power subjects. It is easy to discover the production of new power subjects and the environmental practices through which they acquire power. This process is helpful to reveal the microscopic ecological processes underlying environmental degradation. In addition, this framework breaks through the traditional dimensions of “social construction of conservation space”, “territorialization and influence” and “conservation discourse” that the current research on the political ecology of protected areas focuses on. Additionally, it places more emphasis on local residents’ cognition of these three processes and how to change the existing power relationship through spatial practices, which is an important clue for the analysis of the accidental conservation output of heritage sites. Unfortunately, there is no specific path for local residents to break down the “sealed space” created by the government and business. Perhaps, as Sandbrook said, the unbalanced power relations in conservation will not disappear and cannot withstand simple intervention [44]. 

## Figures and Tables

**Table 1:** Local residents’ perception of conservation.

Questions	Scores of Each Village				
	Qingkou	Gaocheng	Dayutang	Quanfuzhaung	Bada
The government attaches great importance to rice terraces	3.00	1.94	3.63	3.68	3.80
The government attaches great importance to the maintenance and renovation of villages	2.67	2.18	3.94	3.77	2.60
The government attaches great importance to the management of ditches and forests	2.72	2.41	3.81	3.70	3.36
The government attaches great importance to traditional customs	2.67	2.88	3.50	2.91	2.52
After becoming a World Heritage site, economic income increased	3.44	3.02	3.63	2.95	3.04
With heritage status, there are more opportunities to work locally than ever before	3.17	2.53	3.81	2.95	2.80
Are you satisfied with the benefits brought by the development of the heritage area?	3.56	2.59	3.44	2.84	2.80
Do you feel that the economic compensation or income is basically equivalent to the contribution made to heritage protection and development?	3.39	3.00	3.31	2.61	2.40
Are you satisfied with the management of “community + scenic area integrated development” of the tourism enterprise?	4.23	2.25	3.37	2.91	2.66

## Data Availability

Not applicable.
